# A Portrait of Cord Blood Units Distributed for Transplantation from Canadian Blood Services’ Cord Blood Bank: First Analysis

**DOI:** 10.3390/curroncol29120752

**Published:** 2022-12-06

**Authors:** Gaganvir Parmar, Meagan Green, Karen Mostert, Tiffany Lawless, Nicholas Dibdin, Jason Weiss, Kathy Ganz, Tanya Petraszko, Matthew D. Seftel, David S. Allan

**Affiliations:** 1 Stem Cells, Canadian Blood Services, Ottawa, ON K2E 8A6, Canada; 2 Faculty of Medicine, University of Toronto, Toronto, ON M5S 1A8, Canada; 3 Department of Medicine, Faculty of Medicine, University of British Columbia, Vancouver, BC V1Y 1T3, Canada; 4 Department of Medicine and Biochemistry, Microbiology & Immunology, Faculty of Medicine, University of Ottawa, Ottawa, ON K1H 8M5, Canada; 5 Ottawa Hospital Research Institute, Ottawa, ON K1H 8L6, Canada

**Keywords:** cord blood banking, hematopoietic, transplantation, ethnicity

## Abstract

Background: The Canadian Blood Services Cord Blood Bank (CBS CBB) was created to improve access to stem cell products for transplantation for patients across ethnic groups. An analysis of distributed units is needed to assess the effectiveness of the bank to meet the needs of patients from different ethnic groups. Methods: A descriptive analysis was performed on all cord blood units distributed from the CBS’ CBB as of 30 June 2022. Results: Distribution of the first 60 units based on CBS’ CBB inventory has been linear over time. A similar proportion of cord blood unit (CBU) recipients were pediatric or adult. More than half of the cord blood units (56.7%) were distributed to recipients outside of Canada, and CBUs were used to treat a broad range of hematologic and immune disorders. 43.3% of distributed CBUs were of non-Caucasian ethnicity and 18% were from donors self-reporting as multi-ethnic. The mean total nucleated cell counts and total CD34^+^ cell counts were 1.9 ± 0.1 × 10^9^ cells and 5.3 ± 0.5 × 10^6^ CD34^+^ cells, respectively. CD34^+^ cells per kg (recipient weight) varied significantly between pediatric (age 0–4), adolescent (age 5–17) and adult recipients (age 18 and older) (3.1 ± 0.5, 1.4 ± 0.5 and 0.9 ± 0.07 × 10^5^ CD34^+^ cells/kg, respectively). HLA matching was 6/6 (15%), 5/6 (47%) or 4/6 (38%). Conclusions: The CBS’ CBB has facilitated the utilization of banked units for patients across a broad range of ages, geographic distribution, ethnicity, and diseases. Distributed units were well matched for HLA alleles and contained robust cell counts, reflecting a high-quality inventory with significant utility.

## 1. Introduction

Allogeneic hematopoietic cell transplantation (HCT) remains a curative approach to treat patients with hematological malignancies and inherited immune and metabolic disorders [1]. With declining fertility rates, fewer and fewer patients have matched sibling donors [2] and accessing a human leukocyte antigen (HLA)-compatible unrelated donor remains a challenge for many patients. In particular, several non-Caucasian ethnic groups have more complexity in HLA haplotype frequencies which reduces match likelihoods and, furthermore, disproportional representation of some ethnic groups within the global inventory of unrelated donors and cord blood units further reduces the chances of finding a matched donor [3]. Given Canada’s broad ethnic diversity, Canadian Blood Services launched a public cord blood bank to complement donor options within the CBS Stem Cell Registry with a goal of improving access to stem cell donors for all patients and, in particular, patients of non-Caucasian ethnicity.

The Canadian Blood Services’ Cord Blood Bank (CBS’ CBB) was launched in 2013 and has been banking units with total mononuclear cell counts ≥1.5 × 10^9^ and from donors representing a broad range of ethnicities (≥1.3 × 10^9^ for non-Caucasian units). More than 4000 units from the CBS’ CBB are currently searchable in the global inventory of public banks and more than 60% of units are from donors who self-report as non-Caucasians, including many donors reporting multiple ethnic associations [4]. Encouraging HLA-match likelihoods were demonstrated within the bank’s inventory in a recent modelling analysis [4]. Despite reports of success in high-risk leukemia patients [5], cord blood use has fluctuated in recent years in particular due to the increasing use of haploidentical transplantation [6,7]. Usage patterns from the CBS’ CBB have not previously been reported. Moreover, the degree to which usage has been impacted by critical points in the evolution of the bank, such as accreditation by the Association for the Advancement of Blood & Biotherapies (AABB) and the Foundation for the Accreditation of Cellular Therapy (FACT), and utilization during the ongoing pandemic remains unknown. Understanding characteristics of units distributed from the CBS’ CBB with regard to ethnicity is important for the continued efforts to expand ethnic diversity in the bank. We sought to describe the pattern of usage of distributed CBUs from our bank to better understand trends, and modify recruitment accordingly.

## 2. Methods

### 2.1. Donor, Recipient and CBU Information

Donor, product and recipient information for cord blood transplants coordinated by the CBS Stem Cell Registry as of 30 June 2022 (*n* = 60) were extracted from the CBS electronic database. Inventory dates on all banked CBUs as of 30 June 2022 (*n* = 4197) were extracted from the CBS electronic database. All data were deidentified and aggregated prior to analysis.

Utilization scores (US) were calculated for the first 60 CBUs distributed by the CBS CBB as previously described [8]. In brief, the following equation was used to calculate the utilization score for each distributed CBU: Utilization Score = [exp(−6.736 + 0.192X + 0.040Y)]/[1 + exp(−6.736 + 0.192X + 0.040Y)], where X is the total nucleated cell count (×10^8^) and Y is the total CD34^+^ count (×10^6^).

### 2.2. Statistical Analyses

All descriptive statistical analyses were performed using Prism 9 (GraphPad) or Microsoft Excel software. Data are shown as means ± SEM when applicable. All graphs and visuals were generated using either Prism 9 (GraphPad) or Microsoft Excel software.

## 3. Results

**Figure 1:**
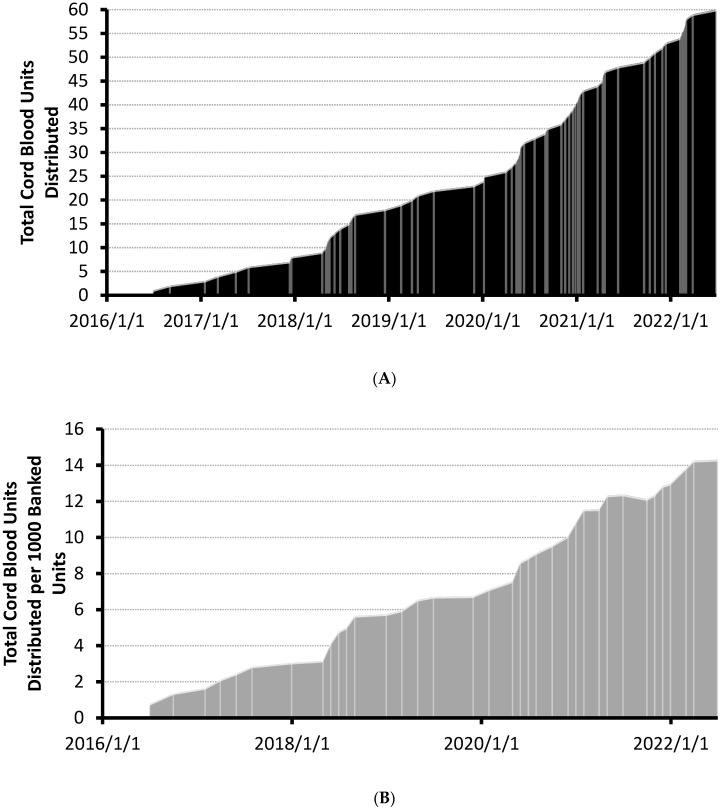
(**A**) Cumulative plot of cord blood units distributed over time. Shipment dates of cord blood units are demarcated with lightly colored lines. Cord blood bank accreditation dates are indicated on the plot. (**B**) Cumulative plot of cord blood units distributed per 1000 banked CBUs over time. Total CBUs distributed were normalized to end-of-month total inventory counts of banked CBUs. Updated total of distributed units per thousand banked CBUs are demarcated with lightly colored lines. Cord blood bank accreditation dates are indicated on the plot. AABB accreditation (1 January 2016) and FACT accreditation (25 February 2019) received.

A total of 4197 units have been banked and were searchable in the CBS’ CBB at the time of analysis. The distribution of the first 60 units increased steadily since the first unit was released in 2016 (see Figure 1A). Cumulative distributions per 1000 banked units increased linearly over a 6 year period (see Figure 1B). No units were distributed before accreditation by the Association for the Advancement of Blood & Biotherapies (AABB) on 1 January 2016 and 19 of the first sixty units (32%) were distributed prior to the NetCord-FACT accreditation on 25 February 2019 (Figure 1A). No apparent change in utilization rates occurred following the FACT accreditation. The rate of total CBU reservation requests also increased linearly over the same time period (Supplementary Figure S1). Annualized gross usage of the CBS’ CBB has steadily increased to 1.4% as of June 2022 (Table 1).

**Figure 2:**
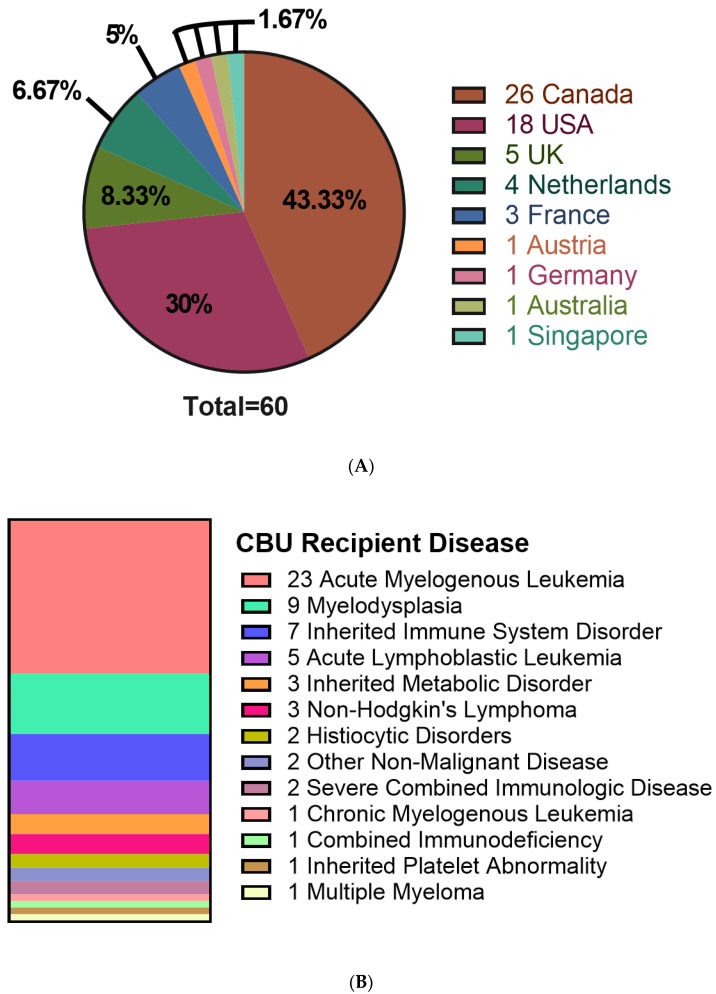
Characteristics of recipients receiving cord blood units from the CBS cord blood bank. (**A**) Location of patients receiving distributed CBUs. (**B**) Disease of patients receiving CBU donations. *N* = 60.

**Figure 3:**
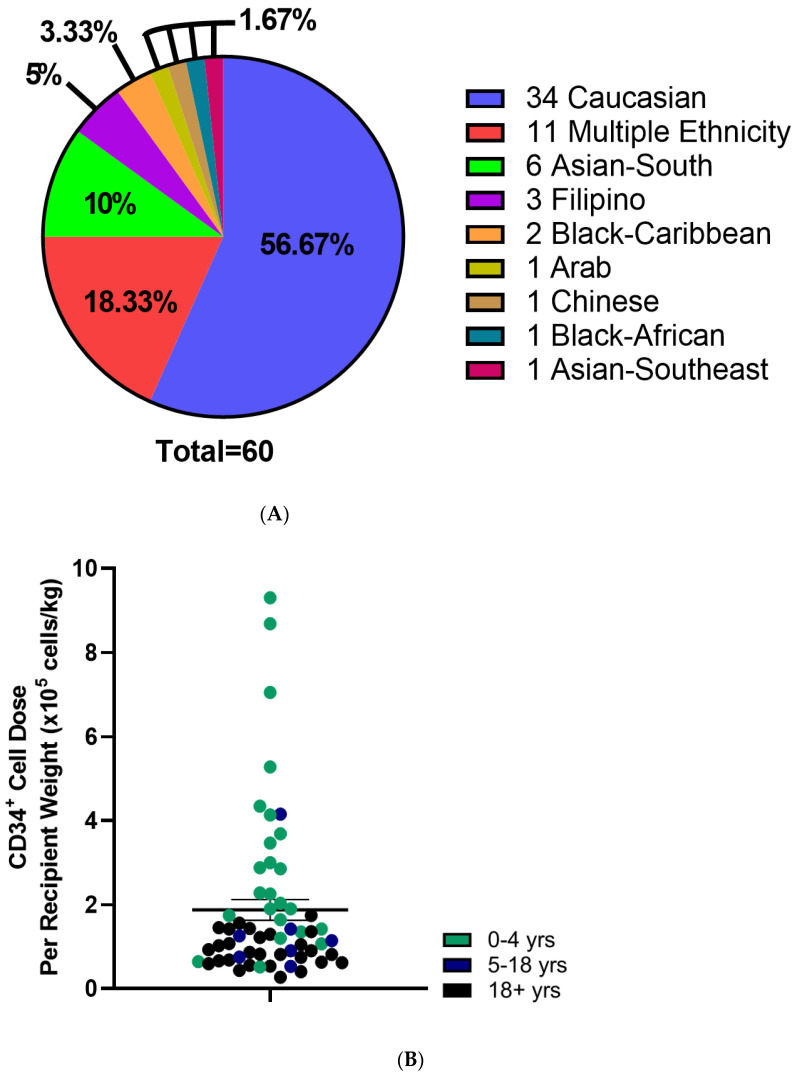
Characteristics of Distributed Cord Blood Units. (**A**) Self reported Ethnicity of distributed CBUs. (**B**) CD34^+^ cell dose per kg recipient weight of distributed CBUs. Patient age indicated with color. *N* = 59. Means indicated ± SEM.

CBU recipients were 18 years or older (46.7%), aged 0–4 years (41.7%) or between 5–17 years of age (11.7%) (Table 2). Regarding the domestic use of units, 43.3% were transplanted into Canadian recipients. International recipients were from the United States (30%), Europe and the UK (23%), Australia (1.7%) and Singapore (1.7%) (Figure 2A). Recipients had a broad range of hematologic and inherited immune and metabolic disorders (Figure 2B). HLA matching was 6/6 (15%), 5/6 (47%) or 4/6 (38%) (Table 2). While the proportion of units from Caucasian donors that were 6/6 HLA matched (7 of 34 units, or 21%) was greater than for non-Caucasian units (2 of 26 units, or 7.9%), the difference was not significant (*p* = 0.28, Fisher’s exact test). Moreover, the effect of ethnicity on HLA match distribution was not significant in a two-way analysis of variance (*p* = 0.16). A total of 10 units (16.7%) were part of a double cord blood transplant and in all 10 cases, the recipients were adults with a hematologic malignancy (Table 2). Donors who self-identified as Caucasian comprised 56.7% of distributed units, while multiethnic donors accounted for 18.3% and other ethnicities were associated with 25% of units (Figure 3A). By contrast, the total inventory of banked units contained 63.5% non-Caucasian units (Figure 3B), including 26% of mothers who self-reported as multiethnic.

Mean total nucleated cell counts and total CD34^+^ cell counts were 1.9 ± 0.1 × 10^9^ cells and 5.3 ± 0.5 × 10^6^ CD34^+^ cells, respectively (Table 3 and Figure 3B). When adjusted to recipient weight, the mean CD34^+^ cell dose was 1.9 × 10^5^ ± 0.2 cells/kg which varied by age (Table 3). Adult recipients (aged 18 and older) received 0.9 ± 0.07 × 10^5^ CD34^+^ cells/kg, while pediatric (ages 0–4 years) and adolescent recipients (ages 5–17) received 3.1 ± 0.5 × 10^5^ and 1.4 ± 0.5 × 10^5^ CD34^+^ cells/kg, respectively (Table 3; Figure 3). TNC and CD34^+^ cell counts were not different between units from Caucasian compared with non-Caucasian ethnicity (Supplementary Figure S2). Utilization scores (US) were calculated for the distributed CBUs to evaluate their predicted utility (Supplementary Figure S3) and were >0.01 in all cases with 26.7% of units exceeding a UI of 0.1 (Table 3).

## 4. Discussion

Cord blood units distributed by the CBS’ CBB are well matched for HLA across a broad diversity of ethnic groups and have been used by adult and pediatric patients, both in Canada and abroad. Steady usage of cord blood units may reflect the overall high quality of units in the bank, early accreditation by the AABB and FACT, and the high proportion of units banked from non-Caucasian donors. In particular, usage of donors from multi-ethnic backgrounds may be of particular importance for patients who may not otherwise have compatible donors. Continued banking of units with high cell counts across a broad range of ethnicities appears worthwhile and important.

While the overall inventory size and usage of the bank is modest on a global scale, continued steady usage diverges from global patterns reported recently [6,7], although usage during the COVID-19 pandemic has been variable, with increased usage in 2020 reported by the EBMT [9], and modest decrease in usage reported by the WMDA [10]. Banking of units with high cell counts and enriching the inventory to >60% non-Caucasian units in the CBS’ CBB may have charted a course to greater interest and steady usage at the CBS’ CBB in comparison to some other banks around the world. Many banks did not introduce thresholds for banking until after many units were already banked, or continue to bank all units collected. However, the number of units collected for the CBS’ CBB that did not meet the threshold for banking was impacted by concomitant delayed clamping of the umbilical cord, a practice that became more widespread following the launch of the CBS’ CBB [11]

The World Marrow Donor Association has reported that cord blood banking is well established in the United States and Europe while banking infrastructure remains lacking in many other countries, skewing the global inventory of banked units towards units of Caucasian ethnicity [12]. The Anthony Nolan Cell Therapy Centre has reported that 73% of banked units are from Caucasian donors with 3.1% from mothers of “mixed race” [13]. They suggest that banking of units with lower cell counts from non-Caucasian donors could enrich ethnic diversity within cord bank inventories. Notably, the CBS’ CBB embraced this approach from the outset using a lower TNC threshold for the banking non-Caucasian units and has banked 63% of units from non-Caucasians with 26% of units donated from mothers of multiple ethnicity associations. Multiethnic donors represented a significant proportion of the units distributed from the CBS’ CBB and units from non-Caucasian donors represented nearly half of the units distributed for transplantation. Cord blood transplantation has increased the opportunities for transplantation in patients from non-Caucasian ethnicities [3,14], although certain ethnic groups continue to face challenges in finding suitable units for transplantation [15] and cord blood units donated by non-European donors may be used preferentially for pediatric patients [13]. The CBS’ CBB is committed to structuring the CBU inventory to better serve non-Caucasian patients who comprise an increasing proportion of the Canadian population [16] and face more challenges in finding HLA-matched unrelated adult donors [5].

Over 40% of the CBU transplantations observed were to recipients aged between 0 and 4 years. While cord blood transplantation has demonstrated success in adult populations [6], marrow and peripheral blood stem cells are often preferred for adult recipients due to the higher TNC and CD34^+^ cell counts in products isolated from these methods [6,7]. CBU selection guidelines suggest minimum CD34^+^ cell doses of 1.5 × 10^5^ CD34^+^ cells/kg recipient weight [17] and higher levels for non-malignant conditions [18,19,20,21]. We observed high cell doses in CBUs selected for pediatric patients; however, cell doses for adult patients were more often below this recommended cut-off. In many cases where the cell dose was low, the unit was part of a double cord blood transplant.

The extent to which cord blood transplantation is preferred over other alternative donor sources for HCT remains under study. A recent real-world analysis using data from the CIBMTR [22] suggests that haploidentical peripheral blood transplants may be preferred over double cord blood transplantation in adults with high-risk hematologic malignancies. While an RCT was completed [23], it did not enroll sufficiently to detect a difference in the primary outcome of progression-free survival, but did observe improved overall survival with haploidentical bone marrow transplant compared to double cord blood transplantation using reduced-intensity regimens. With favorable results from cord blood expansion studies [24], more studies will be needed to clarify the precise role of cord blood transplantation in adults and children.

## 5. Conclusions

This first analysis of 60 units distributed by the CBS’ CBB confirms the ability to facilitate HCT for patients in Canada and worldwide, including adult and pediatric patients with a broad range of diseases. Continued banking of high-quality units will ensure the ongoing availability to support patients undergoing HCT. Moreover, as the diversity of Canada and the global population continues to expand and evolve, maintaining an inventory with an evolving ethnic diversity will be needed, and especially units donated from multiethnic donors, to ensure continued access to stem cell products for patients awaiting transplant.

## Figures and Tables

**Table 1:** Usage of the CBS’ Cord Blood Bank.

Year	Total Cords Distributed (Cumulative)	Total Cords in Inventory	Usage (%)
2015	0	664	0
2016	2	1684	0.12
2017	10	2626	0.30
2018	18	3137	0.57
2019	23	3437	0.67
2020	40	3701	1.1
2021	53	4085	1.3
June 2022	60	4197	1.4

**Table 2:** Characteristics of cord blood unit recipients.

Recipient Age, *n* (%), and Weight, kg (Range)	
0–4 years	25 (41.7), 10.6 (4–19)
5–17 years	7 (11.7), 42.7 (13–108)
18+ years	28 (46.7), 84.2 (54–157)
**Recipient Location (*N*, (%))**	
Canada	26 (43.3)
USA	18 (30)
Other	16 (26.7)
**Recipient Disease (*N*, (%))**	
Hematologic Cancer	42 (70)
Inherited Disorder	14 (23.3)
Other	4 (6.7)
**Donor-Recipient Match Level (*N*, (%))**	
6/6 Match	9 (15)
5/6 Match	28 (46.7)
4/6 Match	23 (38.3)
Double cord blood recipients (*N*, (%)) *	10 (16.7)

* All adults (18 years or older).

**Table 3:** Characteristics of distributed cord blood units.

CBU Ethnicity (*N*, (%))	
Caucasian	34 (56.7)
Multiple Ethnicity	11 (18.3)
Other	15 (25)
Total Nucleated Cell Count (×10^9^ cells)	1.9 ± 0.1
Total CD34^+^ Cell Count (×10^6^ cells)	5.3 ± 0.5
Utilization Score	
Below 0.1 (*N*, (%))	44 (73.3)
Above 0.1 (*N*, (%))	16 (26.7)
CD34^+^ Cell Dose (×10^5^ cells/kg recipient weight)	1.9 ± 0.2
0–4 years	3.1 ± 0.5
5–17 years	1.4 ± 0.5
18+ years	0.9 ± 0.07

## Data Availability

The data presented in this study are available on request from the corresponding author.

## Supplementary Materials

The following supporting information can be downloaded at: https://www.mdpi.com/article/10.3390/curroncol29120752/s1, Figure S1: Cumulative plot of cord blood unit reserve requests per 1000 banked CBUs over time; Figure S2: Analysis of total nucleated cells (TNC) and CD34^+^ cells in Caucasian (*n* = 34) and non-Caucasian units (*n* = 26); Figure S3: Utilization scores of the first 60 cord blood units distributed by the Canadian Blood Services Cord Blood Bank.
