# Supplementary material for: A Portrait of Cord Blood Units Distributed for Transplantation from Canadian Blood Services’ Cord Blood Bank: First Analysis
Source: Curr Oncol. 2022 Dec 6;29(12):9572–81. doi: 10.3390/curroncol29120752 (PMC9777040; doi:10.3390/curroncol29120752)

Supplemental Figure S1. Cumulative plot of cord blood unit reserve requests per 1000 banked CBUs over time. Total CBU reservation requests were normalized to end of month total inventory counts of banked CBUs. Updated total of reserve requests per thousand banked CBUs are demarcated with lightly colored lines. Reservations are distinct from distributions

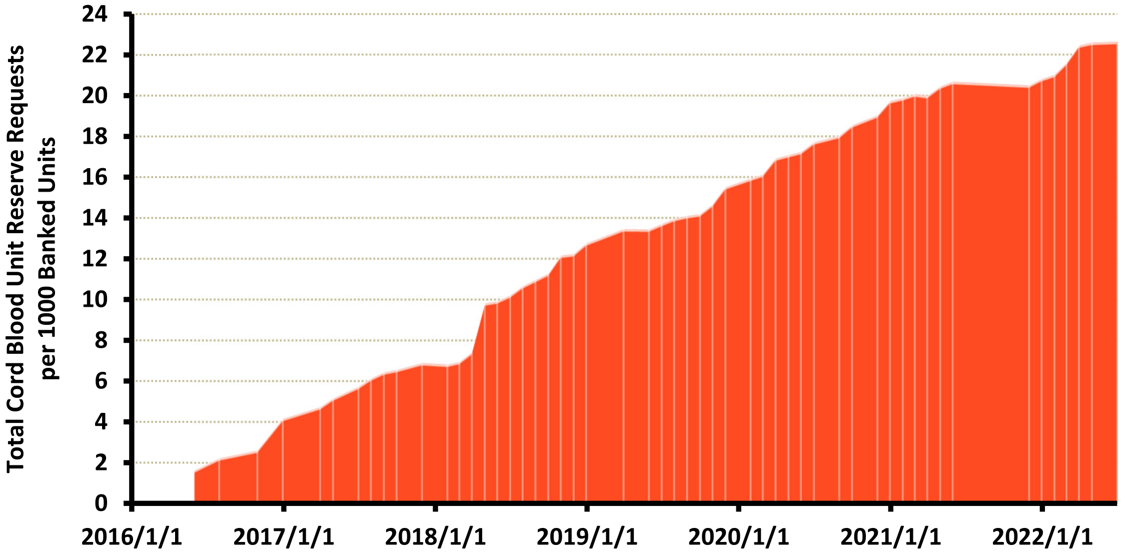

Supplemental Figure S2. Analysis of total nucleated cells (TNC) and CD34+ cells in Caucasian (n=34) and non-Caucasian units (n=26). (A) TNC were 2.0 ± 0.1 and 1.8 ± 0.1 x 10^9 for Caucasian and non-Caucasian units, respectively. **(B)** Total CD34+ cells were 5.2 ± 0.6 and 5.5 ± 0.7 x 10^6 for Caucasian and non-Caucasian, respectively.

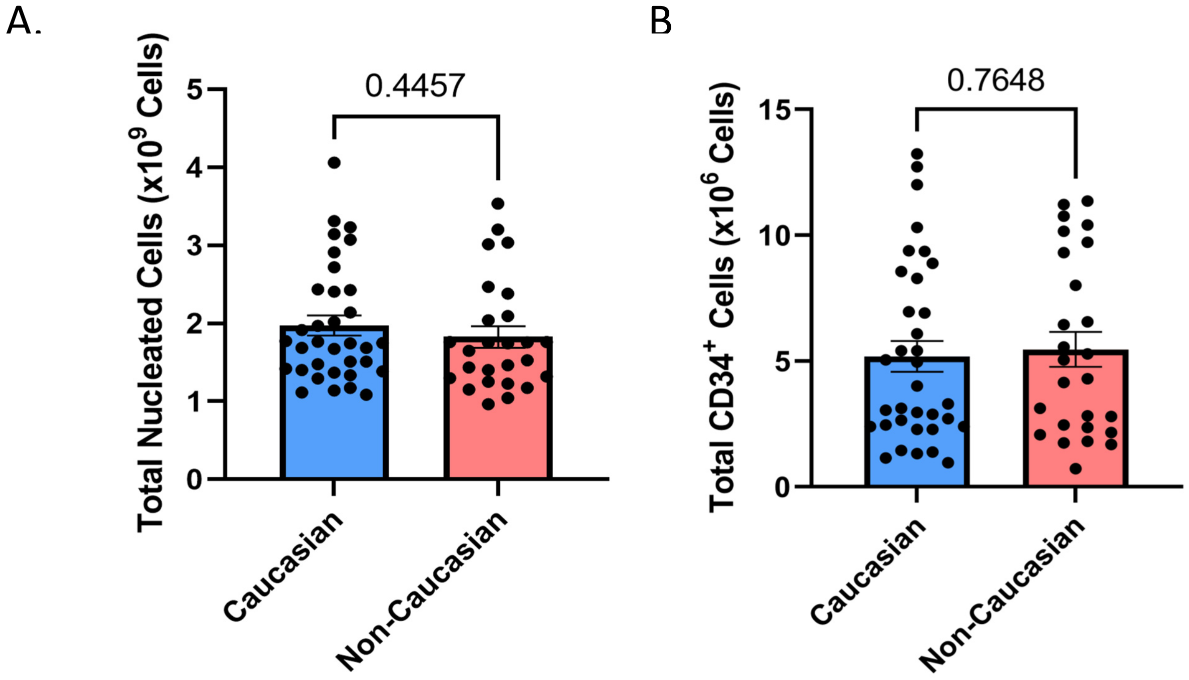

Supplemental Figure S3. Utilization scores of the first 60 cord blood units distributed by the Canadian Blood Services Cord Blood Bank. Utilization scores were calculated as previously described [8]. In brief, the following equation was used to calculate the utilization score for each distributed CBU: Utilization Score = [exp(−6.736 + 0.192X + 0.040Y)] / [1 + exp(−6.736 + 0.192X + 0.040Y)], where X is the total nucleated cell count (×10^8) and Y is the total $CD34^+$ count (×10^6).

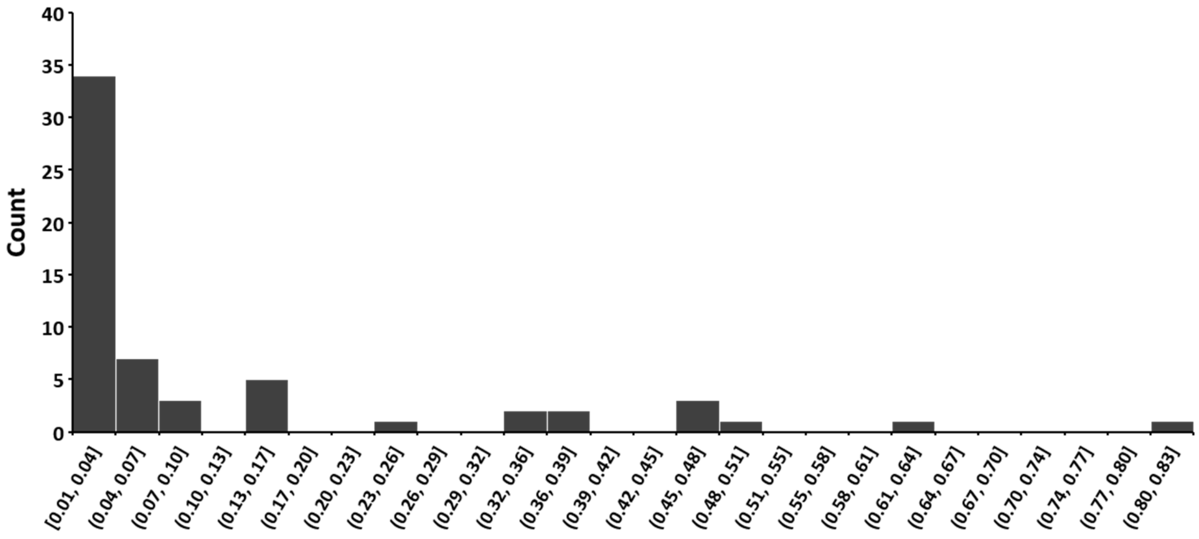

Supplement: Supplementary file 1 [file curroncol-29-00752-s001.zip › curroncol-2019497-supplementary.pdf]
